# Protein profile of basal prostate epithelial progenitor cells—stage‐specific embryonal antigen 4 expressing cells have enhanced regenerative potential *in vivo*

**DOI:** 10.1111/jcmm.12785

**Published:** 2016-02-05

**Authors:** Thomas Höfner, Corinna Klein, Christian Eisen, Teresa Rigo‐Watermeier, Axel Haferkamp, Martin R. Sprick

**Affiliations:** ^1^ Heidelberg Institute for Stem Cell Technology and Experimental Medicine (HI‐STEM gGmbH) Heidelberg Germany; ^2^ Department of Urology University Hospital Frankfurt Frankfurt Germany; ^3^ Division of Stem Cells and Cancer German Cancer Research Center (DKFZ) Heidelberg Germany; ^4^ German Cancer Consortium (DKTK) Heidelberg Germany

**Keywords:** CD13, Syndecan‐1, prostate stem cells, prostate progenitor cells, SSEA

## Abstract

The long‐term propagation of basal prostate progenitor cells *ex vivo* has been very difficult in the past. The development of novel methods to expand prostate progenitor cells *in vitro* allows determining their cell surface phenotype in greater detail. Mouse (Lin^−^Sca‐1^+^ CD49f^+^ Trop2^high^‐phenotype) and human (Lin^−^ CD49f^+^ TROP2^high^) basal prostate progenitor cells were expanded *in vitro*. Human and mouse cells were screened using 242 anti‐human or 176 antimouse monoclonal antibodies recognizing the cell surface protein profile. Quantitative expression was evaluated at the single‐cell level using flow cytometry. Differentially expressed cell surface proteins were evaluated in conjunction with the known CD49f^+^/TROP2^high^ phenotype of basal prostate progenitor cells and characterized by *in vivo* sandwich‐transplantation experiments using nude mice. The phenotype of basal prostate progenitor cells was determined as CD9^+^/CD24^+^/CD29^+^/CD44^+^/CD47^+^/CD49f^+^/CD104^+^/CD147^+^/CD326^+^/Trop2^high^ of mouse as well as human origin. Our analysis revealed several proteins, such as CD13, Syndecan‐1 and stage‐specific embryonal antigens (SSEAs), as being differentially expressed on murine and human CD49f^+^ TROP2^+^ basal prostate progenitor cells. Transplantation experiments suggest that CD49f^+^ TROP2^high^ SSEA‐4^high^ human prostate basal progenitor cells to be more potent to regenerate prostate tubules *in vivo* as compared with CD49f^+^ TROP2^high^ or CD49f^+^ TROP2^high^ SSEA‐4^low^ cells. Determination of the cell surface protein profile of functionally defined murine and human basal prostate progenitor cells reveals differentially expressed proteins that may change the potency and regenerative function of epithelial progenitor cells within the prostate. SSEA‐4 is a candidate cell surface marker that putatively enables a more accurate identification of the basal PESC lineage.

## Introduction

Several model systems have been developed to understand the biological mechanisms involved during benign prostatic enlargement and prostate cancer, the latter being the most common type of cancer in men. It has been suggested that basal epithelial stem/progenitor cells (basal PESCs) are critical for the development of the prostatic gland and that they play an important role in prostate cancer development 1, 2, 3, 4. However, basal PESCs are rare, with a frequency of 1–5% within all prostatic cells, which clearly complicates biological studies using these cells 5, 6. Isolation and *ex vivo* expansion of basal PESCs have been further complicated by their dependence on poorly understood factors supplied by a prostate cell niche composed of smooth muscle cells, fibroblasts, neuroendocrine cells, and differentiating and mature prostate epithelial cells 7. Although significant progress had been made, culture techniques up to now allowed for only limited expansion of prostate epithelial cells (PrECs), which rapidly ceased to proliferate 8, 9, 10. We recently discovered new methods to grow and expand both murine and human basal PESCs in serum‐ and feeder‐free conditions 11. The methods enrich for adherent mouse basal PESCs with a Lin^−^ Sca‐1^+^ CD49f^+^ Trop2^high^ phenotype. Progesterone and sodium selenite are additionally required for the growth of human Lin^−^ CD49f^+^ TROP2^high^ basal PESCs. When transplanted in combination with urogenital sinus mesenchyme (UGSM), expanded mouse and human basal PESCs generate ectopic prostatic tubules, demonstrating their stem cell activity *in vivo* 11. The possible expansion of basal PESCs to significant cell numbers allowed us high‐throughput analyses to characterize their cell surface protein profile in detail.

## Materials and methods

### Adherent expansion of primary murine and human basal PESCs

Murine and human basal PESCs were isolated and propagated as described 11. Microdissection, enzymatic digestion and preparation of single cells from male C57Bl/6 mice were performed as described previously 6. For isolation of primary human cells from surgical prostate tissues, we obtained informed consent according to the principles of the Declaration of Helsinki. Procedures were approved by the responsible ethics committee of Heidelberg University (permit S‐479/2009). Briefly, MACS enrichment for EPCAM+ cells was performed after primary preparation of single‐cell suspensions from murine and human prostates. Magnetic enrichment was performed using the autoMACS Pro Separator (Miltenyi Biotec, Bergisch‐Gladbach, Germany). After magnetic enrichment, murine cells were cultured (adherent) on hydrophobic (suspension) culture flasks (Cellstar; Greiner Bio‐One, Kremsmuenster, Austria), and human cells were cultured on net‐negative pretreated surface flasks (Primaria; BD, Franklin Lakes, New Jersey, USA). The best media for the expansion of murine basal PESCs consists of Advanced DMEM/F12 supplemented with additional glutamine, glucose, EGF, bFGF, LONG R3 IGF‐I, holo‐transferrin and insulin. The best media for the expansion of human basal PESCs is the murine formulation plus additional progesterone and sodium selenite. We previously characterized expanded basal PESCs demonstrating predominant basal cell marker (*e.g*. CK5, p63) expressions within the undifferentiated progenitor culture 11.

### High‐throughput screening of cell surface proteins by flow cytometry

We prepared single‐cell suspensions of 1.5 × 10^8^ cells from each murine and human culture cells using StemPro‐Accutase (Gibco, Carlsbad, California, USA) corresponding to culture passage 7 and 8. Cells were stained in 96‐well plates using BD Lyoplate Cell Surface Marker Screening Panels containing antibodies against 242 human or 176 mouse cell surface antigens, respectively. These panels include the appropriate isotype controls for each antibody used. After washing, cells were resuspended in stain buffer [PBS supplemented with 5 mM ethylenediaminetetraacetic acid (EDTA)] containing propidium iodide for dead cell exclusion. Flow cytometry screening (Alexa 647 signals) was performed using a BD FACS Array Bioanalyzer system, and final analysis was done with FlowJo software (Tree Star, Inc, Ashland, Oregon, USA).

### Sorting and mouse experiments to evaluate *in vivo* stem cell capability

All cell sortings were performed on BD FACS Aria II cell sorter using a 100 μM nozzle. To minimize loss of cell viability, we performed experiments on cell suspensions, prepared shortly before flow cytometry from cultured cells. We detached the cells using StemPro‐Accutase (Gibco). Antibody staining was performed in PBS supplemented with 5 mM EDTA. Prior to flow cytometry or sorting, cells were filtered using 40‐μm filters. The sorting buffer included PBS, 5 mM EDTA and 10 mM ROCK inhibitor (Y‐27632; Tocris Bioscience, Tocris, Bristol, UK). Forward‐scatter height (FSC‐H) *versus* forward‐scatter width (FSC‐W) and side‐scatter height (SSC‐H) *versus* side‐scatter width (SSC‐W) profiles were used to eliminate cell doublets. Dead cells were eliminated by excluding PI+ cells, whereas contaminating human or mouse Lin+ cells were eliminated by gating on Ter119/CD31/CD45‐FITC for mouse and CD45/CD3‐FITC for human cells. Gates for FACS experiments were determined by using isotype controls for the respective specific antibodies used. Gates were then set to exclude the respective population in the isotype control experiment. All mouse experiments were approved by the animal‐protection officers of the German Cancer Research Center (DKFZ) and in accordance with German law (Approval number, G18‐12). Male nude mice were bred at the animal facility of the DKFZ and maintained under pathogen‐free, individual ventilated‐cage conditions. E16 UGSM was used for coinjections with culture‐derived basal PESCs to provide the necessary growth signals to promote *in vivo* prostate gland regeneration. Before performing the coinjections, UGSM was prepared freshly from foetuses of E16 C57Bl/6 mice as previously described by Lukacs *et al*. 6. Briefly, timed pregnancies of C57/BL6 mice were set up and pregnant females were killed at day 16 (E16). Under the stereomicroscope, the foetus was cut in half, the bottom half placed in a supine position while holding the hind legs apart. The urogenital sinus is connected to the bladder and was removed intact, followed by enzymatic digest of the microdissected mesenchyme with 10× collagenase for 2 hrs. Urogenital sinus mesenchyme cells were washed in PBS (4°C) and filtered with 40 μm pore size before used in coinjections with basal PESCs 6.

### Proof of *in vivo* prostate regeneration by lentiviral gene transfer in expanded PESCs

The LeGO‐V2 (Venus) vector was previously described 12 and kindly provided by Kristoffer Weber and Boris Fehse. Lentiviral particles were generated as previously described 13. For transduction, human basal PESCs were cultured for 24 hrs at a fixed cell number. Target cells were incubated in the presence of 8 μg/ml polybrene for 12 hrs at 37°C with viral supernatant at a multiplicity of infection of 50–60 per vector. Transduction efficiency was validated 48–72 hrs after transduction using FACS. To prove *in vivo* stem cell capability of our culture‐derived cells, we coinjected LeGO‐V2 marked cultured human basal PESCs together with E16 UGSM and Matrigel into male nude mice subcutaneously. To support differentiation, we subcutaneously implanted testosterone pellets (12.5 mg/90‐day release; Innovative Research of America). After 10–12 weeks, we harvested the regenerated s.c. grafts for subsequent analyses. Before conducting histological analyses on fixed tissue, we validated direct Venus fluorescence in freshly dissected s.c. grafts under the fluorescence stereomicroscope. Detection of Venus+ in regenerated prostate tissue (proof of regeneration from transplanted PESCs origin) was done by staining s.c. grafts with a monoclonal antibody against GFP/Venus (ab 290; Abcam, Cambridge, UK) 11.

### Statistical analysis

All data are presented as mean ± S.E.M., comparison between groups was done using non‐parametric Kruskal–Wallis tests. (Graph Pad Prism 5.04, Graph Pad Software, La Jolla, California, USA) was used for statistical analyses.

## Results

### High‐throughput screen identifies the specific cell surface protein profile of murine basal PESCs

Using the recently established method, we could expand murine Sca‐1^+^/CD49f^+^/Trop2^high^ basal PESCs up to 1.5 × 10^8^ cells and investigated their cell surface protein profile using 176 validated monoclonal antibodies (results in Table 1).

**Table 1:**
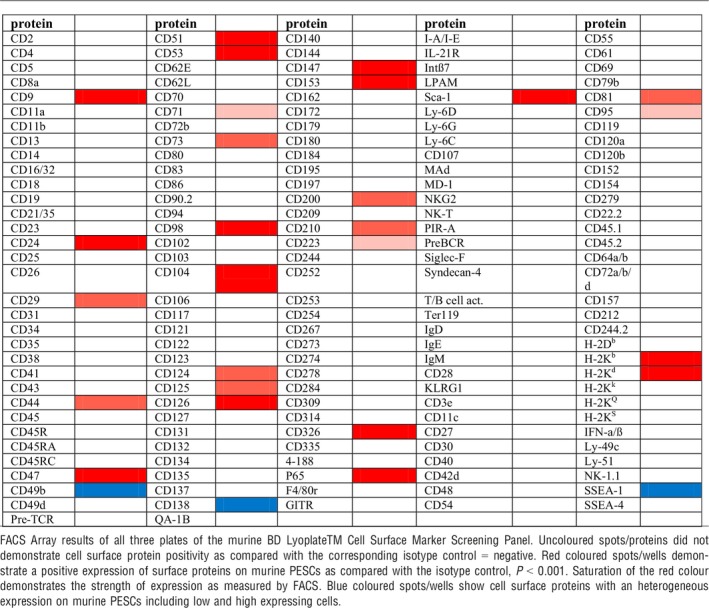
Cell surface protein profile of murine basal PESCs

### High‐throughput screen identifies the specific cell surface protein profile of human basal PESCs

We next performed this screen with *ex vivo* expanded human CD49f^+^/TROP2^high^ basal PESCs using 242 validated monoclonal antibodies (Table 2). Staining of murine and human cells revealed that basal PESCs, in addition to their expression CD49f^+^/TROP2^high^, are positive for a variety of additional markers (Tables 1 and 2). Both mouse and human basal PESCs share the CD9^+^/CD24^+^/CD29^+^/CD44^+^/CD47^+^/CD49f^+^/CD104^+^/CD147^+^/CD326^+^/Trop2^high^ lineage. Table 3 illustrates the cell surface proteins that are well conserved or differentially expressed among both species.

**Table 2:**
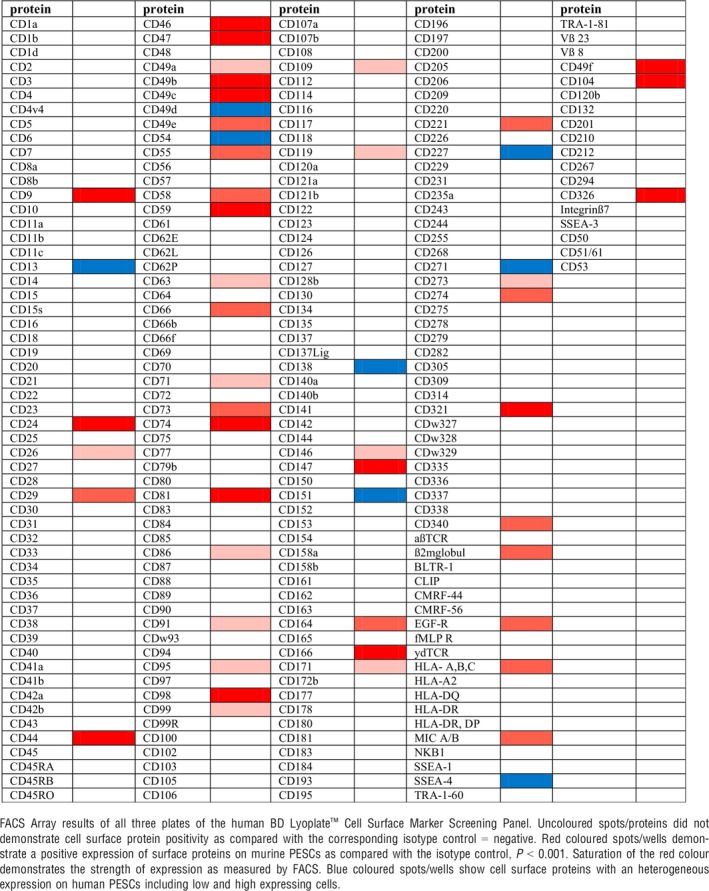
Cell surface protein profile of human basal PESCs

**Table 3:** Cross‐species comparison of surface proteins expressed by basal PESCs

Protein	Expressed in mice	Expressed in humans	Expressed in both species
CD9			
CD13			
CD24			
CD26			
CD29			
CD44			
CD46			
CD47			
CD49b			
CD49c			
CD49d			
CD49e			
CD49f			
CD51			
CD53			
CD54			
CD55			
CD58			
CD59			
CD63			
CD66			
CD71			
CD73			
CD74			
CD81			
CD86			
CD91			
CD95			
CD98			
CD99			
CD104			
CD109			
CD119			
CD124			
CD125			
CD126			
CD138			
CD146			
CD147			
CD151			
CD153			
CD164			
CD166			
CD171			
CD200			
CD210			
CD221			
CD223			
CD227			
CD271			
CD273			
CD274			
CD321			
CD326			
CD340			
β2‐microglobulin			
Sca‐1			
H‐2K^b^			
H‐2K^d^			
HLA‐ A,B,C			

Proteins positively expressed in flow cytometry (FACS Array) as compared with corresponding control, *P* < 0.001.

### Cell surface protein screen identifies CD13 and Syndecan‐1 as being heterogeneously expressed in CD49f^+^/TROP2^high^ human basal PESC

**Figure 1:**
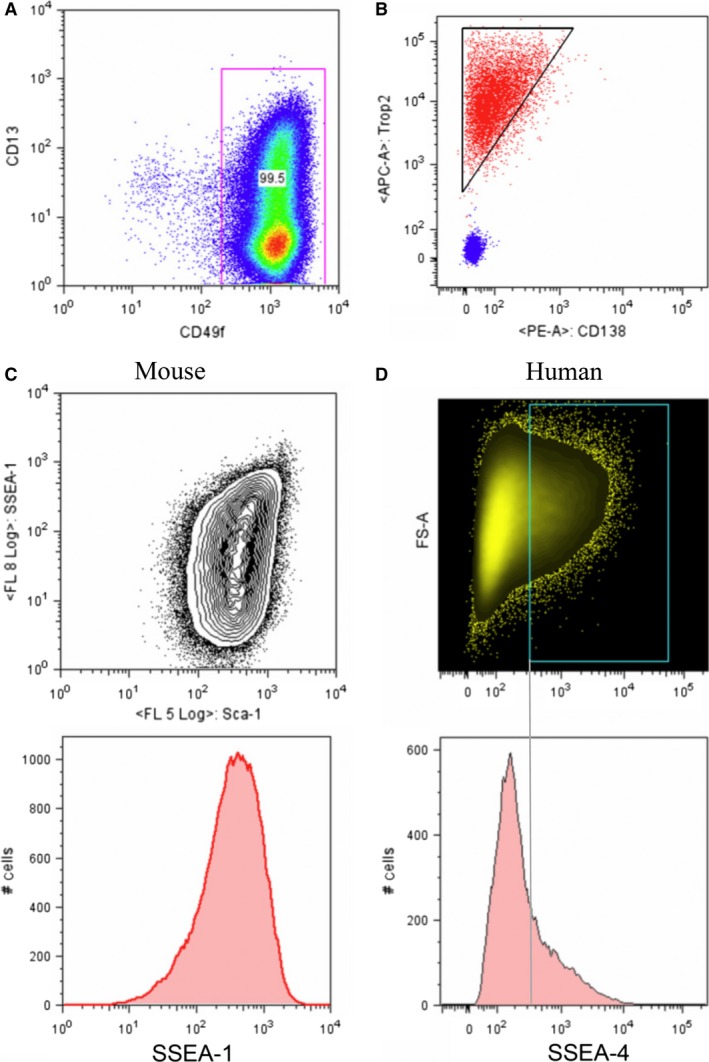
Heterogeneous expression of cell surface proteins on CD49f^+^/TROP2^high^ basal PESCs. (**A**) FACS plot demonstrating the heterogeneous expression of CD13 within CD49f^high^ expressing human basal PESCs, CD13‐APC=clone WN15; CD49f‐PE=clone GoH3, PI ^−^ negative gate, *P* < 0.001 as compared with Mouse IgG1 and Rat IgG2a isotype controls. (**B**) FACS plot demonstrating the heterogeneous expression of CD138 (Syndecan‐1, red colour as compared with isotype control=blue) and the correlation of higher CD138 expression with TROP2 expression in human basal PESCs. CD138‐PE=clone Wi15; TROP2‐APC=clone FAB650A (R&D), PI ^−^ negative gate, *P* < 0.001 as compared with Mouse IgG1 and Mouse IgG2a isotype controls. (**C**) FACS plots demonstrating the heterogeneous expression of SSEA‐1 on murine basal PESCs (below) and the heterogeneous expression of SSEA‐1 in correlation to the Sca‐1^high^ expression of murine basal PESCs. SSEA‐1‐APC=clone MC480; Sca‐1‐PECy7 = clone E13‐161.7, PI ^−^ negative gate, *P* < 0.001 as compared with Mouse IgM and Rat IgG2a isotype controls. (**D**) FACS plots demonstrating the heterogeneous expression of SSEA‐4 on human basal PESCs. Around 20% of all human basal PESCs express SSEA‐4. SSEA‐4‐APC=clone MC813‐70; PI ^−^ negative gate, *P* < 0.001 as compared with Mouse IgG3 isotype control.

By using the cell surface screen, we identified proteins that were differentially expressed within the CD49f^+^/TROP2^high^ human basal PESCs. These data suggest that phenotypically and functionally different cell populations might be contained in the purified basal PESC population. The heterogeneously expressed proteins might be novel markers, identified by such subpopulations. We found that CD13 was heterogeneously expressed in human basal PESCs (Fig. 1A). Additionally, human CD49f^+^/TROP2^high^ basal PESCs show differential Syndecan‐1 (CD138) expression. While 15–50% of all human basal PESCs were negative for Syndecan‐1, the Syndecan‐1 positive fraction was mainly found in the TROP2^high^ cells. CD138 expression levels are positively correlated with that of TROP2 (Fig. 1B).

### Specific heterogeneous expression of stage‐specific embryonal antigens on murine and human basal PESCs

Stage‐specific embryonal antigens (SSEAs) are expressed as carbohydrate adhesion molecules on glycoproteins, glycolipids and proteoglycans of the cell membrane. Stage‐specific embryonal antigens are known and established as markers for murine as well as human embryonic stem (ES) cells 14, 15, 16. Given the observed similarities of gene expression profiles of murine as well as human basal PESCs with the profiles of ES cells 11, we were interested in the specific expression of SSEAs we discovered in the cell surface protein screen. Murine basal PESCs demonstrate a heterogeneous SSEA‐1 expression (Fig. 1C), whereas human basal PESCs do not express SSEA‐1 (Table 2). Overall, 80% of Sca‐1^+^/CD49f^+^/Trop2^high^ cells are positive for SSEA‐1. In contrast, SSEA‐4 was found to be more heterogeneously expressed in human CD49f^+^/TROP2^high^ basal PESCs (Fig. 1D).

### High SSEA‐4 expression marks a distinct population of human CD49f^+^/TROP2^high^ basal PESCs with higher *in vivo* regenerative capacity

**Figure 2:**
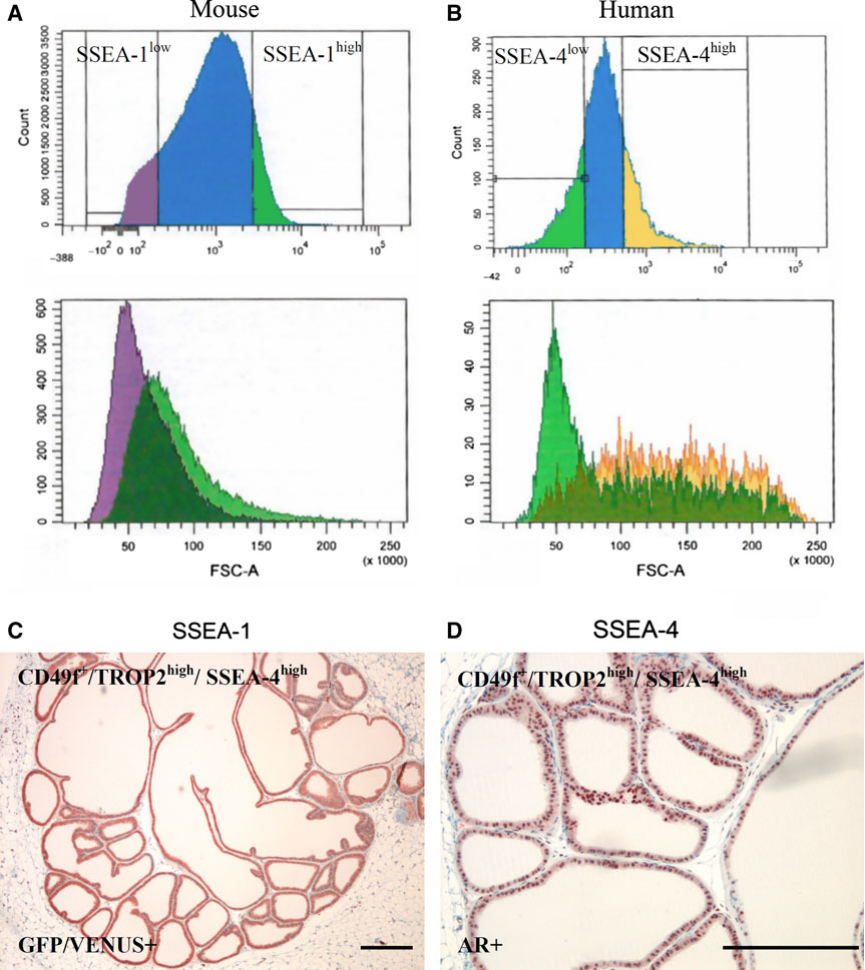
Sorting and *in vivo* transplantation of different SSEA‐expressing basal PESC populations. (**A**) FACS gate of Lin^−^/PI ^−^/Sca‐1^+^/CD49f^+^/Trop2^high^ murine LeGO‐V2‐basal PESCs sorting for SSEA‐1^low^ and SSEA‐1^high^. (**B**) FACS gate of Lin^−^/PI ^−^/CD49f^+^/TROP2^high^ human LeGO‐V2‐basal PESCs sorting for SSEA‐4^low^ and SSEA‐4^high^. (**C**) GFP/Venus positivity of *in vivo* regenerated prostate ducts derived from 250 transplanted human basal PESCs with the sorted Lin^−^/PI ^−^/CD49f^+^/TROP2^high^/SSEA‐4^high^ phenotype, scale bar=500 μm. (**D**) Androgen receptor (AR) positivity of *in vivo* regenerated prostate ducts derived from 250 transplanted human basal PESCs with the sorted Lin^−^/PI ^−^/CD49f^+^/TROP2^high^/SSEA‐4^high^ phenotype, scale bar = 500 μm.

Antigens of the SSEA family have been described to mark undifferentiated ES cells 17. We thus investigated if SSEA expression would also be of functional significance in basal PESCs. We FACS sorted separate populations of LeGO‐V2 transduced murine Sca‐1^+^/CD49f^+^/Trop2^high^/SSEA‐1^low^ and Sca‐1/CD49f^+^/Trop2^high^/SSEA‐1^high^ basal PESCs (Fig. 2A). Comparison of the SSEA‐1^low^ and SSEA‐1^high^ populations in their *in vivo* regenerative potential using s.c. transplantations in nude mice with comparable cell numbers revealed no significant difference (data not shown). Additionally, we FACS sorted populations of LeGO‐V2 transduced human CD49f^+^/TROP2^high^/SSEA‐4^low^ basal PESCs and compared their potential to regenerate prostate gland structures with the sorted CD49f^+^/TROP2^high^/SSEA‐4^high^ population *in vivo* (Fig. 2B). In contrast to the murine SSEA‐1, expression of human SSEA‐4 demonstrates a correlation with the FACS forward scatter (size of cells). The limiting dilution transplantation experiments revealed an increased regenerative capacity of CD49f^+^/TROP2^high^/SSEA‐4^high^ cells to form androgen receptor positive prostatic tubules *in vivo* as compared with the pooled CD49f^+^/TROP2^high^ as well as CD49f^+^/TROP2^high^/SSEA‐4^low^ basal PESC populations (Table 4, Fig. 2C and D).

**Table 4:** *In vivo* regenerative capacity of FACS sorted (lin^−^/PI^−^) human prostate epithelial progenitor cell populations

CD49f^+^/TROP2^high^		CD49f^+^/TROP2^high^/SSEA‐4^low^		CD49f^+^/TROP2^high^/SSEA‐4^high^	
Cells transplanted	Microscopic GFP+ ducts	Cells transplanted	Microscopic GFP+ ducts	Cells transplanted	Microscopic GFP+ ducts
250	+	250	–	250	+
250	–	250	–	250	+
250	–	250	–	250	+
2500	+	2500	–	2500	+
2500	+	2500	–	2500	+
2500	–	2500	–	2500	–
25,000	+	25,000	–	25,000	+
25,000	–	25,000	–	25,000	–
25,000	–	25,000	–	25,000	–

Sorted populations were transplanted s.c. in nude mice together with E16 UGSM as described 11, *P* = 0.01.

## Discussion

Based on a new method to enrich and expand primary basal prostate progenitor cells from murine and human origin, we investigated their surface protein profile. This profile may serve as a reference for future investigations on basal PESCs. We previously demonstrated that *in vitro* expanded basal PESCs retain stem cell activity 11. In addition to the known proteins CD49f and TROP2, we here describe a number of additional antigens (Tables 1 and 2) that are expressed on those cells. This extended surface marker panel might be of use for future studies, especially those using flow cytometry methodologies. Basal PESCs are suggested to be cells of origin of prostatic diseases by being substantially involved in the development of prostate cancer as well as glandular benign prostatic hyperplasia 1, 2, 3, 18. Therefore, the identification of the cell surface marker profile of basal PESCs is helpful for future scientific studies by providing additional markers for identification and purification. For example, both laminin binding integrins, CD49c and CD29, are observed both in mouse and human PESCs. Interestingly, we also find heterogeneous expression of the tetraspanin CD151, which is known to bind to integrins including CD49c and modulate their function. Thus, our data suggest a potential link worth investigating in the future. Among the investigated differentially regulated proteins CD13, CD138 and SSEAs only the glycosphingolipid SSEA‐4 proved to have a functional influence on the regenerative capacity of basal PESCs to form prostatic glands *in vivo*. Glycosphingolipids are a group of lipids that are involved in the formation of cell membranes. They consist of a hydrophobic ceramide portion and a glycosidically linked carbohydrate. This carbohydrate is presented on the outside of the cell membrane, where it is involved in adhesion or signalling between cells 19, 20. The addition of a third sugar molecule to the lactose disaccharide of glycosphingolipids determines the further division into more complex sphingolipids, including the SSEA‐1 and SSEA‐4 investigated in our study. Since the establishment of the first murine ES cell line in 1981, and the demonstration that this cell line specifically expresses glycosphingolipids, glycosphingolipids in particular the SSEAs were established in numerous studies as a marker of pluripotent and multipotent stem cells 17. It has been demonstrated that murine ES cells express SSEA‐1 21. Interestingly, SSEA‐1 is also a marker of the differentiation of murine ES cells. It was demonstrated that approximately 50% of undifferentiated murine ES cells express SSEA‐1; however, less than 10% of the more differentiated cells express SSEA‐1 22. ES cells of monkeys and humans in contrast express SSEA‐3 and SSEA‐4 as markers, but not SSEA‐1 16, 23. Similar to SSEA‐1 in the mouse, human SSEA‐3 and SSEA‐4 expression correlated with the level of differentiation of ES cell lines 24. These species‐specific expressions of SSEAs were confirmed in induced pluripotent stem (IPS) cells. After reprogramming mouse fibroblasts into functional IPS cells, these cells demonstrate positive SSEA‐1 expression 25. In contrast, human IPS cells expressed SSEA‐3 and SSEA‐4, but showed no SSEA‐1 expression (Takahashi *et al*., 2007; Yu *et al*., 2007). Stage‐specific embryonal antigens were also used as markers in adult stem cells as well as for cancer (medulloblastoma) 26. Doetsch *et al*. detected SSEA‐1 in murine adult neural stem cells 27. In addition, SSEA‐4 expression was detected in human adult mesenchymal stem cells within the bone marrow 28.

Our results demonstrate that up to 80% of expanded murine Sca‐1^+^/CD49f^+^/Trop2^high^ basal PESCs express SSEA‐1. On the other hand, 20–30% of expanded CD49f^+^/TROP2^high^ human basal PESCs express SSEA‐4. We thus for the first time describe SSEA expression on adult PESCs. However, sorting and transplanting different populations of SSEA‐1^low^ and SSEA‐1^high^ expressing murine basal PESCs did not result in significant changes in *in vivo* regenerative capacity. In addition, SSEA‐1 was not specific for epithelial cells in the murine prostate, as also 15% of all lineage negative cells expressed SSEA‐1 (data not shown). It is possible that these cells correspond to mesenchymal progenitor cells in the prostate 29, which has to be investigated in future studies. Conversely, we can demonstrate results of a potential superior regenerative stem cell capacity for the human CD49f^+^/TROP2^high^/SSEA‐4^high^ basal PESCs lineage (0.2–0.4% of all prostatic cells) as compared to the known CD49f^+^/TROP2^high^ basal PESCs population (1–3% of all prostatic cells) to form prostate tubules *in vivo*. This result, however, remains provisional as the true stem cell frequency of SSEA‐4^high^ basal PESCs remains undetermined in our study. Nevertheless, we suggest that SSEA‐4 positivity could further narrow down the lineage of basal PESCs towards the true prostate basal epithelial stem cell. Our new methods to expand functional basal PESCs may open up new possibilities for studying the aetiology of prostatic diseases. Discovering the cell surface protein profile of murine and human basal PESCs reveals differentially expressed proteins that may change the biology and regenerative function of these cells within the prostate. Stage‐specific embryonal antigen‐4 is a candidate cell surface marker that putatively enables a more accurate identification of the basal PESC lineage.

## Conflicts of interest

The authors confirm that there are no conflicts of interest.

## Author contribution

T.H. developed the methods and designed the study, performed culture and differentiation experiments *in vitro* and *in vivo* and wrote the article. C.E. helped with lentiviral constructs and performed bioinformatics analyses. C.K. performed experiments and supervised all mouse transplantation experiments. T.R.‐W. helped with experiments. A.H. analysed the data and/or provided intellectual guidance regarding their interpretation. M.R.S. designed the study, analysed and evaluated results, and wrote the article.
